# *HLA-G* 3′ untranslated region variants +3187G/G, +3196G/G and +3035T define diametrical clinical status and disease outcome in epithelial ovarian cancer

**DOI:** 10.1038/s41598-019-41900-z

**Published:** 2019-04-01

**Authors:** Esther Schwich, Vera Rebmann, Rafael Tomoya Michita, Hana Rohn, Jan Willem Voncken, Peter A. Horn, Rainer Kimmig, Sabine Kasimir-Bauer, Paul Buderath

**Affiliations:** 1 Institute for Transfusion Medicine, University Hospital Essen, University of Duisburg-Essen, Virchowstr. 179, 45147 Essen, Germany; 2 Genetics Department, Post-Graduation Program in Genetics and Molecular Biology, Universidade Federal do Rio Grande do Sul (UFRGS), Porto Alegre, Brazil; 3 Department of Infectious Diseases, University Hospital Essen, University of Duisburg-Essen, Hufelandstr. 55, 45147 Essen, Germany; 4 Molecular Genetics, Maastricht University, PO Box 6161, 6200 MD Maastricht, Netherlands; 5 Department for Gynecology and Obstetrics, University Hospital Essen, University of Duisburg-Essen, Hufelandstr. 55, 45147 Essen, Germany

## Abstract

Expression of the non-classical human leukocyte antigen-G (HLA-G) promotes cancer progression in various malignancies including epithelial ovarian cancer (EOC). As single nucleotide polymorphisms (SNPs) in the *HLA-G* 3′ untranslated region (UTR) regulate HLA-G expression, we investigated *HLA-G* 3′UTR haplotypes arranged by SNPs in healthy controls (n = 75) and primary EOC patients (n = 79) and determined soluble HLA-G (sHLA-G) levels. Results were related to the clinical status and outcome. Although haplotype frequencies were similar in patients and controls, (i) sHLA-G levels were increased in EOC independent of the haplotype, (ii) homozygosity for UTR-1 or UTR-2 genotypes were significantly associated with metastases formation and presence of circulating tumor cells before therapy, whereas (iii) the UTR-5 and UTR-7 haplotypes were significantly associated with a beneficial clinical outcome regarding negative nodal status, early FIGO staging, and improved overall survival. Lastly, (iv) the ambivalent impact on clinical EOC aspects could be deduced to specific SNPs in the *HLA-G* 3′UTR: +3187G, +3196G and +3035T alleles. Our results give evidence that even if the genetic background of the *HLA-G* 3′UTR is identical between patients and controls, certain SNPs have the potential to contribute to diametrical clinical status/outcome in EOC.

## Introduction

The human leukocyte antigen G (HLA-G), a non-classical human leukocyte antigen, belongs to the immune checkpoint molecules that regulate/control immune effector responses. The HLA-G molecule is associated with anti-inflammatory and immune-modulatory properties through interaction with inhibitory receptors such as immunoglobulin-like transcript (ILT)2, ILT4 and killer-cell immunoglobulin-like receptor (KIR)2DL4 expressed on different immune-competent cells^1^. HLA-G inhibits B cells, T cells, and natural killer cells, and induces regulatory T cells thereby mediating escape from the host immune surveillance^2^. In contrast to classical HLA molecules, HLA-G displays limited allelic variations, but exists in seven isoforms due to alternative splicing^3^. These isoforms can be expressed at the cell surface (HLA-G1, -G2, -G3 and -G4)^4^ or as secreted molecules (HLA-G5, -G6, and -G7)^5,6^. Soluble forms can be released by healthy cells e.g. trophoblasts, adult and embryonic stem cells, and monocytes^7^ or by malignant cells including breast cancer^8,9^, melanoma^7^, renal cancer^10^, and ovarian cancer cells^9,11^.

Physiologically, HLA-G cell surface expression is restricted to the maternal-fetal interface in placental mammals, where it mediates immune tolerance, and to immune privileged adult tissues^12^. In contrast, neo-ectopic or aberrant expression of HLA-G and its soluble forms has been associated with a vast variety of pathological situations^13^. In the context of malignancies, HLA-G has been implicated in cancer invasiveness and metastatic progression such as epithelial ovarian cancer (EOC)^2,14,15^, the most lethal of gynecologic malignancies^16^. EOC is classified into four surgical stages according to the International Federation of Gynecology and Obstetrics (FIGO); this classification considers the extent of ovary affection, the extent of spreading outside the ovaries and outside the pelvis^16^. The microenvironment of EOC provides a driving force for cancer cell invasion and metastasis^17^. Although most EOC initially respond to primary treatment, tumor recurrence is frequently not limited to drug-resistant tumors, resulting in an impaired overall patient survival (OS)^18^. Dissemination of single tumor cells (DTCs) into the blood-stream plays a major role in drug resistance and thereby disease recurrence and metastases formation in EOC^19^. Likewise, the presence of circulating tumor cells (CTCs) negatively correlates with OS in EOC patients^20,21^.

HLA-G is located on chromosome 6p21.3 and is composed of eight exons and seven introns, and so far 58 HLA-G allelic variations have been identified^22^. Importantly, the regulation of HLA-G expression and of its soluble forms encompasses post-transcriptional processes among which alternative splicing, altered mRNA stability, microRNA-mediated protein expression and impaired protein transport to the cell surface^2^. Especially the polymorphic 3′untranslated region (UTR) shared by the HLA-G1 to HLA-G6 transcripts (~370 bp) plays a pivotal role in HLA-G expression by interfering with transcription, splicing, mRNA stability and translation^23^. Here, sixteen single nucleotide polymorphisms (SNP; +3001C/T, +3003C/T, +3010C/G, +3027C/A, +3032C/G, +3035C/T, +3052C/T, +3092G/T, +3111A/G, +3121C/T, +3142C/G, +3177G/T, +3183A/G, +3187A/G, +3196C/G, and +3227A/G) and a 14 bp insertion/deletion (INS/DEL) located at position +2961 have been identified in the 3′UTR potentially modifying the affinity of gene targeted sequences for post-transcriptional factors^24^. These polymorphisms arrange as haplotypes, named UTRs (Supplementary Info 1). UTR-1 to UTR-8 and UTR-18 are the most frequent ones^22,25^. Six already identified microRNA (miR), miR-148a, miR-148b, miR-152, miR-133a, miR-628-5p, and miR-548q, have been reported to bind to certain SNPs in the 3′UTR in a sequence-specific manner, leading to downregulation of HLA-G expression^22^. Particularly, UTR-7, encompassing the three most studied variations (14 bp INS, +3142G, and +3187G), has been reported to correlate with lower plasma levels of sHLA-G^26^. Moreover, many types of cancer exhibit an aberrant miR expression profile^27,28^, which modulates the tumor microenvironment via non-cell-autonomous mechanisms^28^ and is involved in tumor initiation, progression, metastasis formation and therapy resistance^29^. We here propose that, in analogy with other clinical disorders^30,31^, variable *HLA-G* 3′UTR sites in combination with the cellular microenvironment may be critical in influencing HLA-G expression.

In this retrospective study we (i) defined the *HLA-G* 3′UTR haplotypes from primary patients with serous ovarian carcinoma, (ii) determined the association of the genetic background with the presence of CTCs, and (iii) analyzed the impact of the genetic background on clinical status and disease outcome of EOC.

## Results

### *HLA-G* 3′UTR haplotypes in EOC patients and healthy controls

To define the 3′UTR haplotypes in EOC patients (n = 79) and healthy donors (HD; n = 75), we sequenced and analyzed 15 previously described polymorphic variations in the *HLA-G* 3′UTR. As expected, a high linkage disequilibrium (LD) was observed among the SNPs showing a minor allele frequency (MAF) >1%. In addition, a nearly perfect LD (D′ = 0.97, r^2^ = 0.92) was observed between +3010C/G and +3142G/C alleles in healthy women (Supplementary Data 1). Overall, 9 out of 16 different haplotypes were identified with a frequency >1% and were considered in this study (Supplementary Data 2). In line with previous data^26,32^, UTR-1 (29% and 28%) and UTR-2 (29% and 27%) displayed the most abundant haplotypes in EOC patients and healthy women, respectively. Interestingly, one undesignated UTR haplotype (UTR-undes.) differing from UTR-2 only at position +3142 by a C instead of a G (Supplementary Info 1), was exclusively identified in the patients’ group with a frequency of 3%. Statistically significant differences between the two study groups were not observed for the genotype or haplotype distribution (Supplementary Data 3).

### sHLA-G in EOC patients and healthy controls

**Figure 1:**
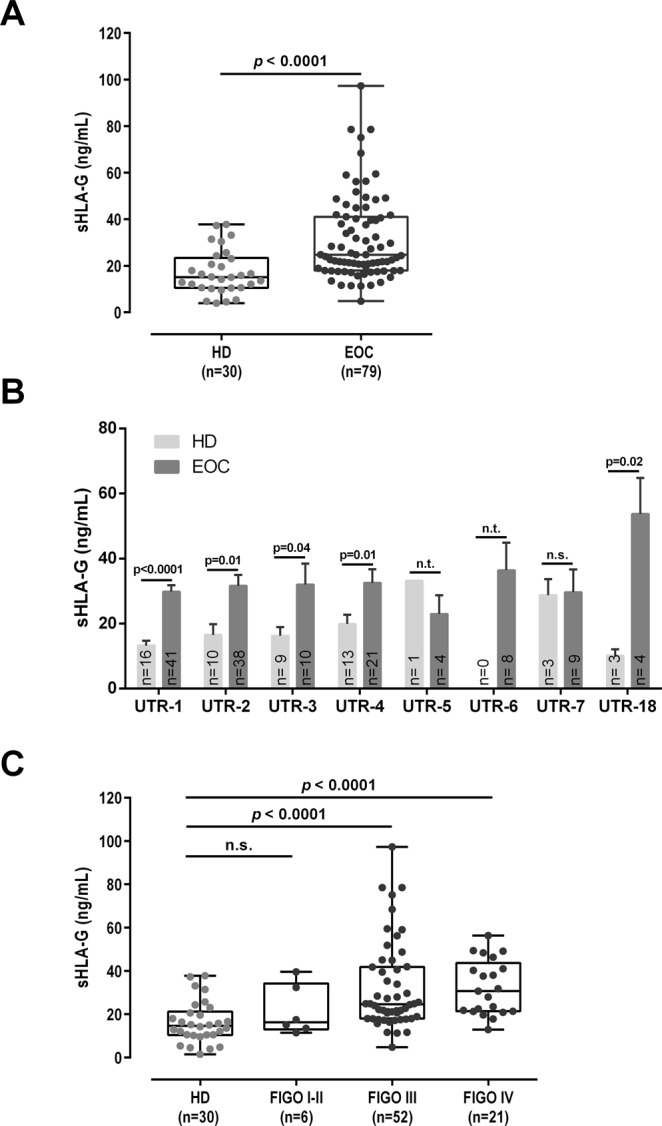
Comparison of sHLA-G levels of EOC patients and healthy donors. (**A**) sHLA-G is significantly (p < 0.0001) elevated in EOC compared to healthy donors (HD). (**B**) Elevated sHLA-G levels in EOC are independent of a specific *HLA-G* 3′UTR haplotype. Bars indicate mean value ± SEM. (**C**) sHLA-G levels increase with ascending FIGO stage in EOC patients without reaching significance. sHLA-G levels are given in ng/ml. Statistic was performed by Mann-Whitney test (**A**,**C**), or Kruskal-Wallis with Dunn’s test for multiple comparison (**B**). n.s. not significant, n.t. not tested due to low numbers.

Mean sHLA-G levels were almost two-fold elevated in EOC patients compared to HD (n = 30; p < 0.0001), independent of the patients’ UTR haplotype (Fig. 1a,b). In EOC patients, median sHLA-G levels increased with ascending FIGO stages without reaching significance (Fig. 1c). Among EOC patients, the nodal status, metastasis formation, presence of CTC and DTC prior to therapy and OS and PFS was not associated with sHLA-G levels (data not shown).

### *HLA-G* 3′UTR haplotypes UTR-1 or UTR-2 are associated with metastases and presence of CTCs in EOC patients

**Figure 2:**
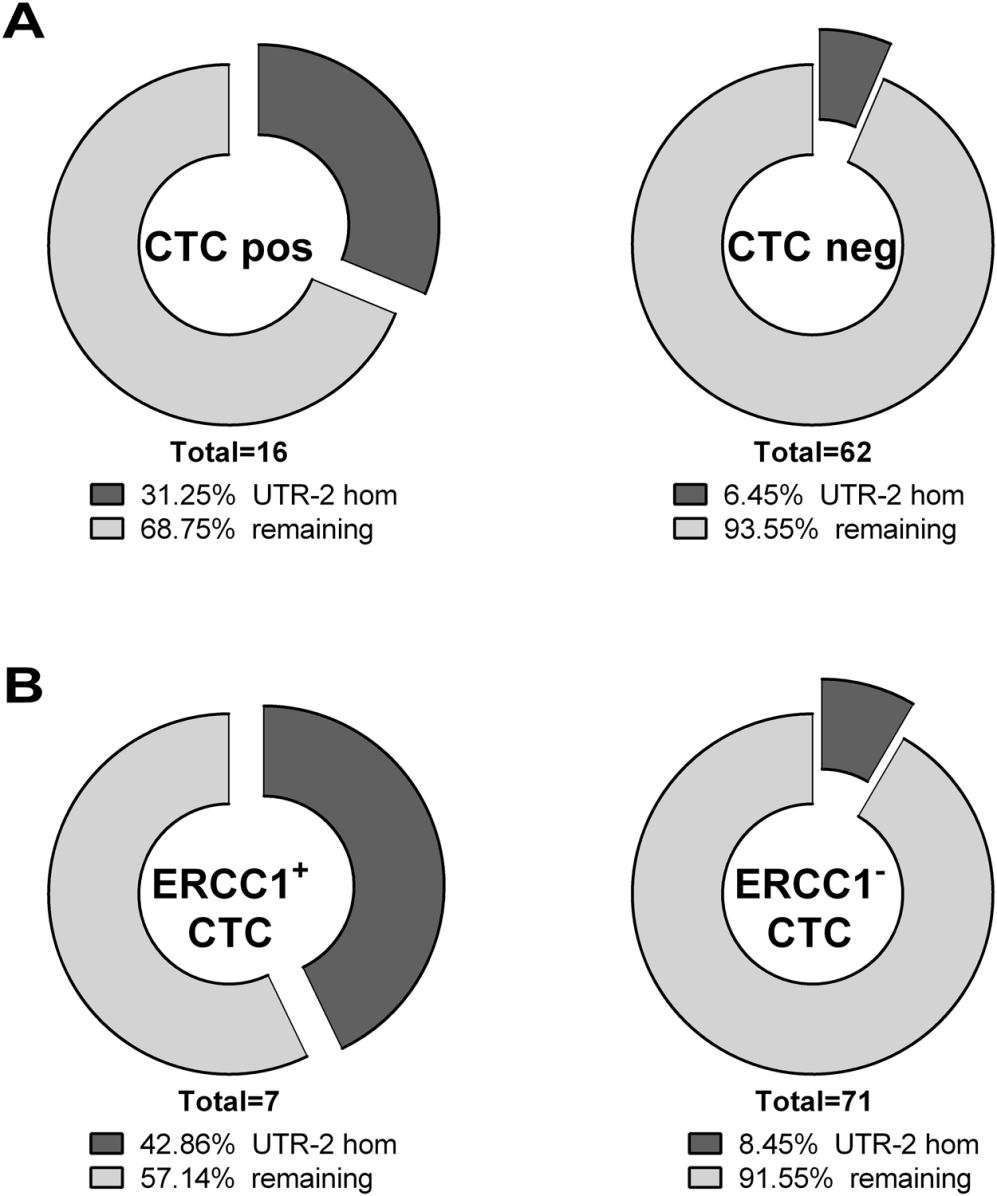
Association of HLA-G UTR-2 haplotype with presence of CTCs, especially with ERCC1+ CTCs before therapy. (**A**) Distribution of homozygous UTR-2 carriers compared to the remaining haplotypes in association with CTCs. CTCs before therapy could not be determined for one patient. (**B**) Distribution of homozygous UTR-2 carriers compared to the remaining haplotypes in association with ERCC1+ CTCs. ERCC1 gene expression analysis could not be determined for one patient.

UTR haplotype analysis showed that allelic homozygosity for UTR-1 was significantly associated with the presence of metastases (*p* = 0.0044, Odds Ratio (OR): 17.81, 95% Confidence Interval (CI): 1.94–163.7), whereas presence of UTR-5 was positively associated with a negative nodal status (*p* = 0.0392, OR: 0.066, 95% CI: 0.00–1.361) and early FIGO staging (*p* = 0.001, OR: 0.014, 95% CI: 0.00–0.176; Table 1). Homozygous UTR-2 genotype was significantly associated with the presence of CTCs before therapy (*p* = 0.0151, OR: 6.59, 95% CI: 1.52–28.51; Table 2 and Fig. 2). Interestingly, concerning CTC specificity, the latter one is significantly associated with ERCC1^+^ CTCs (*p* = 0.0312, OR: 8.00, 95% CI: 1.44–44.47). None of the UTR haplotypes or genotypes were associated with CTCs after therapy (data not shown).

**Table 1:** *HLA-G* 3′UTR haplotypes are associated with the clinical status of EOC patients.

	Metastasis formation	M_1_^b^	M_0_^b^	*p* ^a^	OR (95% CI)
UTR-1	UTR-1/UTR-1	5	1	**0**.**0044**	**17**.**81** (**1**.**94**–**163.7**)
	Remaining	16	57		
	**Nodal status**	**pN**_**1**_^**b**^	**pN**_**0**_^**b**^	***p*** ^**a**^	**OR** **(95% CI)**
UTR-5	positive	0	3	**0**.**0392**	**0**.**066** (**0**.**00**–**1.361**)
	negative	33	15		
	**FIGO**	**III-IV** ^**b**^	**I-II** ^**b**^	***p*** ^**a**^	**OR** **(95% CI)**
UTR-5	positive	1	3	**0**.**001**	**0**.**014** (**0.00**–**0.176**)
	negative	72	3		

CI – confidence interval; M_0_ – no metastasis formation; M_1_ – metastasis formation; pN_0_ – no nodal infestation; pN_1_ – nodal infestation; OR – odds ratio.

^a^p-values were calculated by GraphPad Prism using Fisher’s exact test, alpha <0.05.

^b^Numbers reflect cases.

**Table 2:** Homozygous UTR-2 of the *HLA-G* 3′UTR is associated with the presence of CTCs, in particular ERCC1 positive CTCs, before therapy.

	UTR-2 hom^b^	remaining^b^	*p* ^a^	OR (95% CI)
CTC pos	5	11	**0**.**0151**	**6**.**591** (**1**.**52**–**28.51**)
CTC neg	4	58		
MUC^+^ CTC	3	7	n.s.	4.429 (0.90–21.75)
MUC^−^ CTC	6	62		
ERCC1^+^ CTC	3	4	**0**.**0312**	**8**.**000** (**1**.**44**–**44.47**)
ERCC1^−^ CTC	6	65		
EPCAM^+^ CTC	1	4	n.s.	2.031 (0.20–20.50)
EPCAM^−^ CTC	8	65		
HER2^+^ CTC	0	1	n.s.	2.404 (0.09–63.40)
HER2^−^ CTC	9	68		

CI – confidence interval; CTC – circulating tumor cells; EPCAM – epithelial cell adhesion molecule; ERCC1 – Excision Repair cross-complementing group 1; HER-2 – human epidermal growth factor 2; hom – homozygous; MUC – Mucin; n.s. – not significant; OR – odds ratio.

^a^p-values were calculated by GraphPad Prism using Fisher’s exact test, alpha <0.05.

^b^Numbers reflect cases.

### HLA G 3′UTR SNP genotypes +3187G/G or +3196G/G are associated with metastases and presence of CTCs and DTCs in EOC patients

Allelic variation at position +3187 distinguished UTR-1 from the others by carrying a G instead of an A (Supplementary Info 1). Interestingly, the +3187GG genotype was significantly associated with metastasis formation (*p* = 0.0044, OR: 17.81, 95% CI: 1.94–163.7; Table 3). Concerning UTR-2 genotype, homozygous +3196G variant, being exclusively present in UTR-2 and in UTR-undes. in our study, was strongly associated with the presence of both, CTCs and DTCs before therapy (*p* = 0.0075, OR: 6.84, 95% CI: 1.75–26.76, and *p* = 0.0156, OR: 5.84, 95% CI: 1.40–24.27, respectively; Table 3). None of the SNP variants were associated with CTCs after therapy (Supplementary Data 4). These observations suggest an association of specific *HLA-G* 3′UTR-1 and 2 genotypes with oncological phenotypes. Association between the remaining SNPs and the clinical parameters were not found (Supplementary Data 4).

**Table 3:** Single nucleotide polymorphisms in the UTR-1 and UTR-2 haplotypes of the *HLA-G* 3′UTR are associated with aggravated clinical status in EOC patients.

		M_1_^b^	M_0_^b^	*p* ^a^	OR (95% CI)
+3187	GG	5	1	**0**.**0044**	17.81 (1.94–163.7)
	AA or AG	16	57		
		**CTC pos** ^b^	**CTC neg** ^b^	***p*** ^**a**^	**OR** **(95% CI)**
+3196	GG	6	5	**0**.**0075**	6.840 (1.75–26.76)
	CC or CG	10	57		
		**DTC pos** ^b^	**DTC neg** ^b^	***p*** ^**a**^	**OR** **(95% CI)**
+3196	GG	8	3	**0**.**0156**	5.841 (1.40–24.27)
	CC or CG	21	46		

CI – confidence interval; CTC – circulating tumor cells; DTC – disseminated tumor cells; M_0_ – no metastasis formation; M_1_ – metastasis formation; neg – negative; OR – odds ratio; pos – positive; ^a^p-values were calculated by GraphPad Prism using Fisher’s exact test, alpha <0.05.

^b^Numbers reflect cases.

### *HLA-G* 3′UTR haplotypes UTR-5 or UTR-7 are associated with early FIGO staging and improved clinical outcome in EOC patients

**Figure 3:**
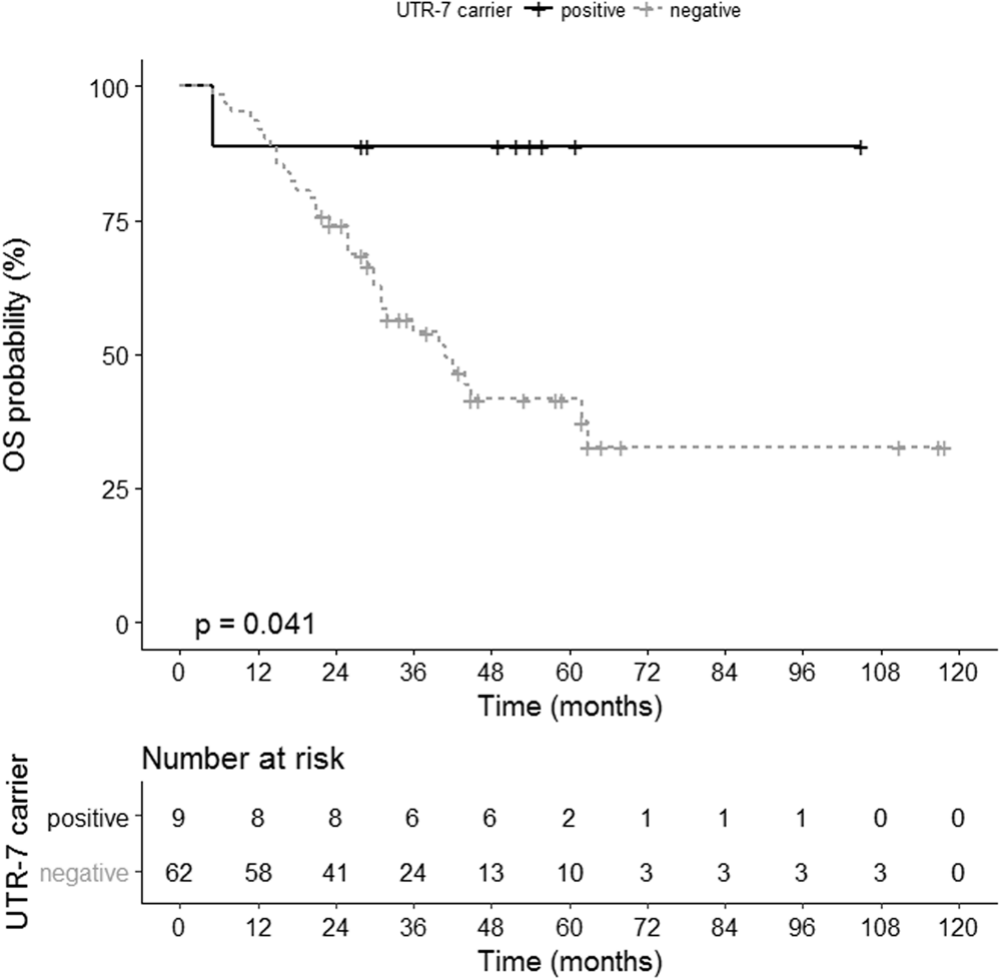
Association of UTR-7 haplotype of HLA-G with an improved OS in EOC patients. Kaplan-Meier plot of the OS of EOC patients carrying UTR-7 (n = 9) and patients not carrying UTR-7 (n = 62).

Regarding disease classification, UTR-5 seemed to be a prognostic beneficial factor in EOC, as it was significantly associated with early FIGO: Three out of six (50%) EOC patients classified as FIGO I-II carried UTR-5, whereas only one out of 73 patients (7,7%) with advanced FIGO (III-IV) stages carried UTR-5 (*p* = 0.001, OR: 0.01, 95% CI: 0.00–0.18; Table 1). Similarly, all patients with nodal infestation were UTR-5 negative (n = 33), whereas in three out of 18 patients without nodal infestation UTR-5 was present (*p* = 0.0392, OR: 0.06, 95% CI: 0.00–1.36; Table 1). Assessment of OS by combined Kaplan-Meier analysis and Log-rank testing revealed that exclusively patients carrying UTR-7 (n = 9) had a significantly prolonged OS with an undefined median survival time compared to the UTR-7 negative patients (n = 62) with a median survival time of 41 ± 7 months (*p* = 0.041, HR: 0.17, 95% CI: 0.14–0.95; Fig. 3). None of the UTR haplotypes were associated with PFS.

### *HLA-G* 3′UTR SNP variant +3035T is associated with improved clinical outcome in EOC patients

**Figure 4:**
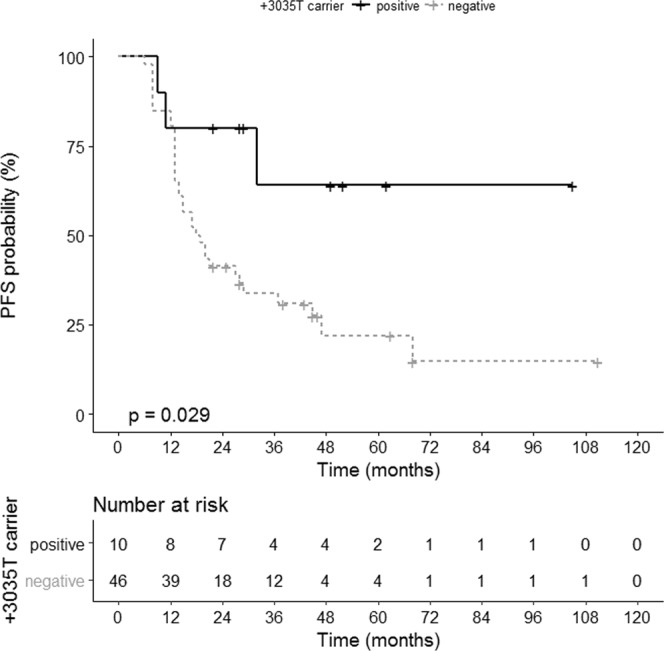
Association of *HLA-G* 3′UTR SNP variant +3035T with an improved PFS in EOC patients. Kaplan-Meier plot of the PFS of EOC patients bearing the +3035T variant (n = 10) and patients not carrying +3035T (n = 46).

**Figure 5:**
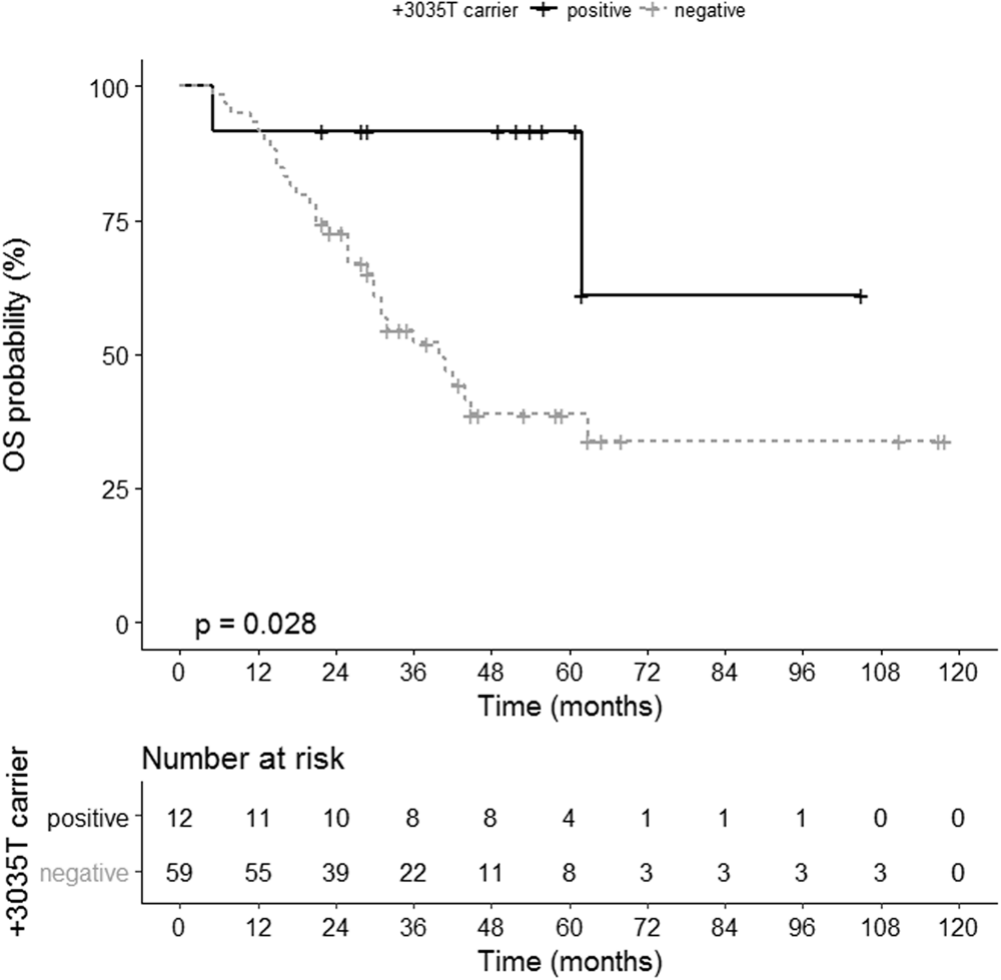
Association of *HLA-G* 3′UTR SNP variant +3035T with an improved OS in EOC patients. Kaplan-Meier plot of the OS of EOC patients bearing the +3035T variant (n = 12) and patients not carrying +3035T (n = 59).

Both, the UTR-5 and the UTR-7 haplotype exclusively harbor a T instead of a C at position +3035 of the *HLA-G* 3′UTR (Supplementary Info 1). Taken this SNP variant into consideration, the +3035T carriers (n = 12) revealed a significantly improved PFS (*p* = 0.029, HR: 0.30, 95% CI: 0.19–0.90; Fig. 4) and OS (*p* = 0.028, HR: 0.23, 95% CI: 0.17–0.90; Fig. 5) compared to non-T carriers with an undefined median survival time compared to 40 ± 6 and 18 ± 3 month, respectively. These findings suggest that UTR-5 and UTR-7 genotypes are beneficial prognostic factors in EOC, as they appear to significantly associate with early FIGO and enhanced OS, respectively. The +3035T in both UTR-5 and -7 independently associates with PFS and OS in EOC.

## Discussion

HLA-G expression is considered as an important immune escape mechanism of cancer cells^33^. Its expression is regulated at transcriptional, co- and post-transcriptional levels. Regulation at the co- and post-transcriptional level occurs through alternative splicing and/or binding of certain miR within the *HLA-G* 3′UTR, respectively^27^. As HLA-G and sHLA-G are reported to be highly expressed in ovarian cancer^9,15,34,35^, we here examine a possible association between specific *HLA-G* 3′UTR haplotypes and clinical status of EOC, and whether specific SNPs within the *HLA-G* 3′UTR region may have prognostic value with regards to EOC treatment and survival. We demonstrate for the first time (i) that haplotype frequencies of the *HLA-G* 3′UTR between healthy controls and EOC patients are comparable although sHLA-G levels of patients were significantly increased compared to HD independent of their *HLA-G* 3′UTR haplotype, (ii) that homozygosity for UTR-1 or UTR-2 genotypes are significantly associated with metastases formation and presence of CTCs before therapy, (iii) that the UTR-5 and UTR-7 haplotypes are associated with a beneficial clinical outcome, with regards to negative nodal status, early FIGO staging and improved OS, and lastly (iv) that certain allelic variants, among which +3187G, +3196G and +3035T in the *HLA-G* 3′UTR have ambivalent impact on clinical aspects of EOC.

Our comparative haplotype analysis between healthy controls and EOC patients revealed that the overall frequencies of both cohorts are very similar to the ones found in worldwide population studies^25,26,32^. A study of haplotype distribution between papillary thyroid cancer and healthy controls demonstrated significant differences^23^. As we did not observe such differences in *HLA-G* 3′UTR distribution, this may point to distinct contribution of HLA-G genotypes to the onset of specific forms of cancer or oncological phenotypes. Although UTR-1 and UTR-2 frequencies encompass more than 50% of all haplotypes, the presence of homozygous status of UTR-1 or UTR-2 is rare. Of note, we found that the frequency of homozygous UTR-1 or UTR-2 was nearly doubled in EOC compared to healthy controls. Although this difference did not reach significance given the current population size, this finding could imply that these genotypes may favor susceptibility to EOC. Indeed, among EOC patients, these genotypes were associated with adverse cancer status: Homozygous UTR-1 genotype is associates with metastases, which may relate to different immune-suppressive microenvironment of advanced CTC compared to the primary tumors. Interestingly, in EOC a cluster of specific miRs has been functionally connected to metastatic lesions^36^. So far, UTR-2 has not been associated with metastasis by other studies; however a relationship with an increased risk of neurotoxicity after chemotherapy treatment in colorectal cancer was observed^37^. We^20,38^ and others^39^ have already described CTCs as a negative prognostic marker regarding OS as well as chemotherapy resistance in EOC^19^. Of note, in the present study the detection of CTCs before therapy was associated with a homozygous UTR-2 genotype. In particular, homozygous UTR-2 status in EOC patients appears to be related to the presence of ERCC1 positive CTC subpopulation. In this context, high ERCC1 expression has recently been linked to a worse PFS^21^ and OS among EOC patients, especially in late stage and poor differentiation serous ovarian patients^21,40^. Recently, we showed that auxiliary assessment of ERCC1-transcripts expanded the phenotypic spectrum of CTC-detection and we defined a sub-fraction of CTCs^41^. To the best of our knowledge the current study is the first to demonstrate an association between the presence of CTCs and *HLA-G* 3′UTR polymorphisms in EOC. Nevertheless, further studies will have to evaluate whether CTCs express HLA-G allowing their survival in the periphery.

In sharp contrast to the UTR-1/2 haplotypes, our study revealed that carriers of the *HLA-G* 3′UTR haplotypes UTR-5 and UTR-7 have a clinical benefit with respect to long-term OS and negative nodal status. Both haplotypes are associated with a lower HLA-G production compared to UTR-1^26^ thereby preventing HLA-G-mediated immune escape. *In vitro* studies combining site-directed mutagenesis of *HLA-G* 3′UTRs with reporter assays demonstrated that 3′UTR polymorphisms have the potential to affect mRNA stability or degradation via RNA-binding proteins and miRs: again, low HLA-G production was previously associated with UTR-5 and UTR-7, whereas UTR-1 was related to high production^22^. Strikingly, both, UTR-5 and UTR-7, are the only identified haplotypes carrying the rare +3035T variant, which shows to be positively associated with a prolonged OS as well as PFS and being different from all other haplotypes. miR-1224-5p has been reported to bind with high affinity to +3027C/+3035T^42,43^, which is characteristic for the UTR-5 haplotype. Additionally, miR-187 binds with moderate affinity to the +3027C/A/+3035T haplotype^42^, targeting both, the *HLA-G* 3′UTR haplotypes UTR-5 and UTR-7. Our finding that the rare +3035T variant which only occurs in UTR-5 and -7 haplotypes and the +3027A variant which is exclusive for the UTR-7 haplotype, positively associate with increased PFS and OS, is in support of a potential role for sequence-dependent macromolecular control of HLA-G mRNA biology. In line with this notion, +3027A polymorphism was shown to be an independent prognostic factor in pediatric Hodgkin’s lymphoma^44^.

Hitherto, the most studied SNP of the *HLA-G* 3′UTR are a 14 bp INS/DEL and +3142G/C. Although not consistent, the majority of studies have associated the14bp DEL as well as +3142C with high HLA-G production potentially promoting an immune-suppressive environment in various malignancies^23,45–47^. Importantly, the sHLA-G levels of the EOC patients in our study were significantly increased independent of the *HLA-G* 3′UTR haplotype implying that the tumor burden is the main source for the systemic release of sHLA-G molecules. This concept is supported by the fact that increasing sHLA-G levels were found to be associated with ascending FIGO stage preferentially mirroring the extent of cancer. The +3187G as well as +3196G variants in UTR-1 and UTR-2, respectively, were associated with aggravated clinical outcome considering presence of CTC, DTC and metastasis formation. Homozygous +3187G carriers have already been linked to a worse prognosis in both DFS and OS in colorectal cancer^48^. However, to deduce this association to the biologic function, it is mandatory to analyze the expression of HLA-G isoforms, the corresponding miRs, and long non-coding RNAs affecting the binding affinity to the *HLA-G* 3′UTR^49^ in CTC, DTC and metastases. Although no miR binding sites have been defined yet for +3187G/A and +3196C/G SNPs in the Brazilian population^42^, it is conceivable that in the context of EOC, these SNPs represent binding sites for as yet unknown *HLA-G*-targeting miRs. Here, a novel *in silico* analysis of miR targeting the *HLA-G* 3′UTR would be of great interest. Alternatively, such nucleotide variants may affect chemical epigenetic modification of mRNAs^50^. Of note, these two SNPs are in close proximity to the recognized AU-rich motif sequence, which influences alternative pre-mRNA processing, HLA-G mRNA stabilization and, translation (initiation, efficiency) progress and alternative pre-mRNA processing^42,51^. Only two patients (3%) expressed UTR-undes., both of whom had a homozygous genotype. Hitherto, the 14 bp INS has always been associated with the presence of +3142G and +3187A, which correlates to low HLA-G levels. UTR-undes., however, encompasses the 14 bp INS in combination with +3142C and +3187A. Interestingly, +3142G is suggested to increase the binding of miR-148a, miR-148b, and miR-152, suggesting that the allelic variation +3142C found in our cohort of patients might be disadvantageous by impairing binding of regulatory miRs regulatory control in the *HLA-G* 3′UTR. This is also in line with recent studies, in which the +3142CC genotype was shown to be associated with increased HLA-G levels and susceptibility to cancer^23,52^. Further, it already has  been shown that some miRs bind to non-polymorphic sequences of the *HLA-G* 3′UTR in a stable and specific manner, while others bind to polymorphic sequences. Such findings indicate that *HLA-G* co- and post-transcriptional regulation of mRNA might be depending on both, binding of miR and additional RNA-binding protein factors (RBPs) and is subject to both genetic (variants), as well as epigenetic and microenvironmental control to certain variants present and the miR microenvironment^43^. In this context, a recent study of renal cell carcinoma analyzed HLA-G transcript, protein and miR expression pattern, infiltrating immune cells, and clinical outcome: A strong post-transcriptional gene regulation of HLA-G by miR-152, -148A, -148B and -133A has been observed^53^. Immunohistochemical staining revealed an inverse expression of miR-148A and -133A with the HLA-G protein *in situ* and *in vitro* indicating a direct interaction with HLA-G regulatory miRs and the *HLA-G* 3′UTR. Stable miR overexpression caused a downregulation of HLA-G protein enhancing cytotoxic function of immune cells. The association of HLA-G expression with infiltrating cells in HLA-G positive tumors revealed higher numbers of CD3+ and CD8+ T cells but not NK and CD4+ T cells. However, the latter was related to a better disease-specific survival. Concerning ovarian cancer, so far only one study placed emphasis on the analysis of the HLA-G expression in primary tumor lesions and metastatic tissue. In this study it was shown that HLA-G was more frequently expressed in metastatic cells than in primary tumor lesions and the expression of HLA-G inversely associated with the frequency of tumor infiltrating immune cells^54^. Besides other parameters both, the HLA-G expression and the low infiltration of immune cells contributed to a worse prognosis and overall survival^54^. Unfortunately, no *HLA-G* 3′UTR typing was performed in this study to link the increased HLA-G expression in metastatic lesions to a certain *HLA-G* 3′UTR SNP.

## Conclusion

We define multiple different *HLA-G* 3′UTR haplotypes relating to diametrical clinical status and/or disease outcome in EOC patients. We propose that even if the genetic background of the *HLA-G* 3′UTR is identical between physiologic and pathologic conditions, presence of certain SNPs within important regulatory sequences in the *HLA-G* 3′UTR have the potential to contribute to an adverse or beneficial clinical status in EOC. It is worthy to mention that the association of individual SNPs potentially reveals the impact of specific haplotypes on EOC outcome (i.e., at least for +3187G/G and +3196G/G being exclusive for UTR-1 and UTR-2, respectively) due to high LD in *HLA-G* 3′UTR. In addition, the impact of such genetic variants is likely dependent on the interaction with the tumor microenvironment and tumor phenotype phenotype and tumor heterogeneity, which is partly characterized by the mutational burden. Of note, certain mutations have been associated with a worse clinical outcome in high-grade serous ovarian cancer, e.g. CHEK2^55^. As certain *HLA-G* 3′UTR SNPs are associated with a worse clinical outcome, here, *HLA-G* 3′UTR typing or HLA-G expression in tumor lesions may improve the prediction of clinical outcome and thus, the disease management. The present data highlight the complexity of the genetic background of *HLA-G* affecting the clinical course of EOC. Future studies should be aimed at defining the interactome (ncRNAs as well as protein factors) of *HLA-G* 3′UTRs. A comprehensive study combining the aspects of *HLA-G* 3′UTR polymorphisms, HLA-G transcript, protein and miR expression pattern, infiltrating immune cells in primary tumor tissue and metastatic lesions with the clinical outcome of patients would greatly contribute to the understanding of HLA-G mediated tumor immune escape. Improved understanding of *HLA-G* regulation will contribute to the development of strategies modulating its expression and to optimizing the design of (immune-) therapeutic strategies for the treatment of tumor patients. Enlarging the cohorts of women will strengthen the associations found in the present study.

## Methods

### Patients’ characteristics

A total of 79 patients diagnosed between 2001 and 2014 at the Department of Gynecology and Obstetrics, University Hospital Essen, with the histologically confirmed primary diagnosis of EOC were analyzed. All patients received the standard treatment consisting of cyto-reductive surgery and adjuvant platinum-based chemotherapy. Patients with EOC stage FIGOIIIB or higher (18 out of 79 patients) received adjuvant bevacizumab treatment. Patients who did not undergo adjuvant platinum-based chemotherapy as well as patients with secondary malignant diseases were excluded from the analysis. Clinical characteristics of the patients are documented in Table 4. 75 healthy female donors (HD) served as control panel for genotyping. Written informed consent was obtained by all participants and the study was approved by the Local Ethics Committees (Essen 05-2870 and 17-7859) and was performed according to the declaration of Helsinki.

**Table 4:** Patient characteristics at time of primary diagnosis.

Total		n = 79 (%)
Age	Median: 61 (27–88)	
FIGO stage	I-II	6 (**8**%)
	III	52 (**66**%)
	IV	21 (**26**%)
Nodal status	N_0_	18 (**23**%)
	N_1_	33 (**42**%)
	Unknown	28 (**35**%)
Metastases formation	M_0_	58 (**73**%)
	M_1_	21 (**27**%)
Tumor grading	I-II	30 (**38**%)
	III	49 (**62**%)
CTC pos^a^	Before therapy	16/78 (**21**%)
	After therapy	10/28 (**36**%)
DTCs pos^b^	Before therapy	29/78 (**37**%)
Recurrence	No relapse	23 (**29**%)
	Relapse	42 (**53**%)
	Unknown	14 (18%)
Platinum-based chemotherapy	No resistance	51 (**65**%)
	Resistance	13 (**16**%)
	Unknown	15 (**19**%)
Overall Survival	0	40 (**51**%)
	1	36 (**45**%)
	Unknown	3 (**4**%)

CTC – circulating tumor cell; DTCs – disseminated tumor cells; FIGO – Federation of Gynecology and Obstetrics; M_0_ – no metastasis formation; M_1_ – metastasis formation; pN_0_ – no nodal infestation; pN_1_ – nodal infestation; ^a^CTCs before therapy could not be determined for one patients, and CTCs after therapy could not be determined for 51 patients; ^b^DTCs before therapy could not be determined for one patient.

### Sampling of blood

Two times 5 ml ethylenediaminetetraacetic (EDTA) blood were collected for isolation of CTCs before the application of therapeutic substances with an S-Monovette (Sarstedt AG & Co.) and stored at 4 °C until further examination. The samples were processed within 4 hours after blood collection.

### Selection, detection and evaluation of CTCs and DTCs

Enrichment of CTCs and subsequent expression analysis were performed according to Adnatest OvarianCancer (Qiagen, Hilden, Germany). The test has been described in detail^21,41^. Briefly, CTCs were immunomagnetically selected by using the AdnaTest Ovarian Cancer Select targeting epithelial cell adhesion molecule EpCAM, MUC-1 and Mucin-16 (also known as CA-125). Subsequently, RNA was isolated and gene expression analysis was performed by reverse-transcription (RT) and multiplex RT-PCR detecting EpCAM, MUC-1, and CA-125 (AdnaTest Ovarian Cancer Detect). ERCC1-transcripts were investigated in a separate approach by singleplex RT-PCR (AdnaTest Ovarian Cancer Detect). β-actin served as an internal control. DTCs were analyzed as described before^56^. In short, DTCs were analyzed by immunocytochemistry using the pan-cytokeratin antibody A45-B/B3.

### Quantification of soluble HLA-G components

Soluble HLA-G was quantified as previously described^57^. Plasma samples were used in a dilution of 1:2 in PBS and purified HLA-G5 served as standard reagent. The sHLA-G levels were determined by four-parameter curve fitting. ELISA detection limit of sHLA-G was 0.25 ng/ml.

### *HLA-G* 3′UTR analysis

After genomic DNA extraction using the QIAamp DNA Blood Mini Kit (Qiagen, Hilden, Germany) according to manufacturer’s instructions, *HLA-G* 3′UTR typing was performed by polymerase chain reaction (PCR) as previously described^58^. The PCR products were directly sequenced using the reverse primer GmiRNA in an ABI 3730 XL DNA sequencer (Applied Biosystems, Foster City, CA, USA) with polymer POP-7 performed in LGC Genomics (LGC Genomics, Berlin, Germany). Sequencing reactions were performed by using BigDye™ Terminator v3.1 Cycle Sequencing Kit (Applied Biosystems, Foster City, CA, USA). *HLA-G* polymorphism was assessed by interpretation of chromatogram peaks using the FinchTV software version 1.4.0 (available on http://www.geospiza.com/Products/finchtv.shtml). In total, our approach allowed us to evaluate 15 genetic variants (14 bp INS/DEL, +3001C/T, +3003C/T, +3010C/G, +3027A/C, +3032C/G, +3035C/T, +3052C/T, +3092G/T, +3111A/G, +3121C/T, +3142C/G, +3187A/G, +3196C/G, +3227A/G) encompassing the 3′UTR of the *HLA-G* gene. Linkage disequilibrium (LD) was evaluated by the Haploview software^59^ through inspections of D′ and r² coefficients in EOC, healthy controls and pooled sample. Haplotype phasing from all individuals were assessed by PHASE 2.1 software using default parameters^60^. The consistency of the results was checked through 10 independent runs using different seed values. Haplotypes of each subject were inferred with a probability ranging from 0.948 to 1.0 for all subjects. Assessment of haplotype frequencies across different runs showed highly consistent results. *HLA-G* 3′UTR haplotypes were determined according to previous studies^25,32^.

To verify differences regarding haplotype frequencies among the patients cohort (n = 79) and controls (n = 75), a residuals analysis was performed (Supplementary Data 2).

### Statistical analysis

Statistical analyses were performed by using SPSS 22.0 (SPSS Inc., Chicago, IL, USA) and GraphPad Prism V6.0 software (GraphPad Software, San Diego, CA, USA). Allele and genotype frequencies of polymorphic sites were calculated by using two-sided Chi-square test. Contribution of allelic variants to clinical parameters was evaluated by Fisher’s exact test as indicated in the table legend. Overall survival (OS) and progression-free survival (PFS) analysis was assessed by the method of Kaplan-Meier and compared using log-rank test implemented in the R package *survminer* (version 0.4.0; https://CRAN.R-project.or/package=survminer).

### Ethics approval and consent to participate

All aspects of this study were approved by the local ethics committee of the University Hospital Essen. Written informed consent was obtained from all enrolled patients, and all relevant investigations were performed according to the principles of the Declaration of Helsinki.

## Supplementary information

Supplementary Info 1

Supplementary Dataset 1, Supplementary Dataset 2, Supplementary Dataset 3, Supplementary Dataset 4

## Data Availability

All data in our study are available upon request.
