# Supplementary material for: HLA-G 3′ untranslated region variants +3187G/G, +3196G/G and +3035T define diametrical clinical status and disease outcome in epithelial ovarian cancer
Source: Sci Rep. 2019 Apr 1;9:5407. doi: 10.1038/s41598-019-41900-z (PMC6443684; doi:10.1038/s41598-019-41900-z)
Supplement: Supplementary file 1 — Supplementary Info 1 [file 41598_2019_41900_MOESM1_ESM.pdf]

# *HLA-G* 3' untranslated region variants +3187G/G, +3196G/G and +3035T define diametrical clinical status and disease outcome in epithelial ovarian cancer

Esther Schwich[1], Vera Rebmann[1*], Rafael Tomoya Michita[1,2], Hana Rohn[3], Jan Willem Voncken[4], Peter A. Horn[1], Rainer Kimmig[5], Sabine Kasimir-Bauer[5], Paul Buderath[5]

[1] Institute for Transfusion Medicine, University Hospital Essen, University of Duisburg-Essen, Virchowstr. 179, 45147 Essen, Germany

[2] Genetics Department, Post-Graduation Program in Genetics and Molecular Biology, Universidade Federal do Rio Grande do Sul (UFRGS), Porto Alegre, Brazil

[3] Department of Infectious Diseases, University Hospital Essen, University of Duisburg-Essen, Hufelandstr. 55, 45147 Essen, Germany

[4] Molecular Genetics, Maastricht University, PO Box 6161, 6200 MD Maastricht, Netherlands

[5] Department for Gynecology and Obstetrics, University Hospital Essen, University of Duisburg-Essen, Hufelandstr. 55, 45147 Essen, Germany

| | |
|---|---|
| Esther Schwich | Esther.Schwich@uk-essen.de |
| Vera Rebmann | Vera.Rebmann@uk-essen.de |
| Rafael Tomoya Michita | rafael.michita@gmail.com |
| Hana Rohn | Hana.Rohn@uk-essen.de |
| Jan Willem Voncken | W.Voncken@maastrichtuniversity.nl |
| Peter A. Horn | Peter.Horn@uk-essen.de |
| Rainer Kimmig | Rainer.Kimmig@uk-essen.de |
| Sabine Kasimir-Bauer | Sabine.Kasimir-Bauer@uk-essen.de |
| Paul Buderath | Paul.Buderath@uk-essen.de |

*** Correspondence:**

Vera Rebmann

Institute for Transfusion Medicine
University Hospital Essen
University of Duisburg-Essen
Virchowstr. 179
45147 Essen
Germany
Phone: +49 201 723 4206
Vera.Rebmann@uk-essen.de

**Supplementary Information 1. Sequence comparison of the most frequent haplotypes of the *HLA-G* 3'UTR.**

| | **UTR-1** | **UTR-2/ undes.** | **UTR-5** | **UTR-7** | **UTR-3** | **UTR-4** | **UTR-6/ 18** |
|---|---|---|---|---|---|---|---|
| **+2961** | DEL | INS | INS | INS | DEL | DEL | DEL |
| **+3003** | T | T | T | T | T | C | T |
| **+3010** | G | C | C | C | C | G | G |
| **+3027** | C | C | C | **A** | C | C | C |
| **+3035** | C | C | **T** | **T** | C | C | C |
| **+3142** | C | G/C | G | G | G | C | C |
| **+3187** | **G** | A | A | A | A | A | A |
| **+3196** | C | **G** | C | C | C | C | C |
| **+3227** | G | G | G | G | G | G | G/A |
