# Supplementary material for: HLA-G 3′ untranslated region variants +3187G/G, +3196G/G and +3035T define diametrical clinical status and disease outcome in epithelial ovarian cancer
Source: Sci Rep. 2019 Apr 1;9:5407. doi: 10.1038/s41598-019-41900-z (PMC6443684; doi:10.1038/s41598-019-41900-z)
Supplement: Supplementary file 2 — Supplementary Dataset 1, Supplementary Dataset 2, Supplementary Dataset 3, Supplementary Dataset 4 [file 41598_2019_41900_MOESM2_ESM.pdf]

HLA-G 3' untranslated region variants +3187G/G, +3196G/G and +3035T define diametrical clinical status and disease outcome in epithelial ovarian cancer

Esther Schwich[1], Vera Rebmann[1*], Rafael Tomoya Michita[1,2], Hana Rohn[3], Jan Willem Voncken[4], Peter A. Horn[1], Rainer Kimmig[5], Sabine Kasimir-Bauer[5], Paul Buderath[5]

[1] Institute for Transfusion Medicine, University Hospital Essen, University of Duisburg-Essen, Virchowstr. 179, 45147 Essen, Germany

[2] Genetics Department, Post-Graduation Program in Genetics and Molecular Biology, Universidade Federal do Rio Grande do Sul (UFRGS), Porto Alegre, Brazil

[3] Department of Infectious Diseases, University Hospital Essen, University of Duisburg-Essen, Hufelandstr. 55, 45147 Essen, Germany

[4] Molecular Genetics, Maastricht University, PO Box 6161, 6200 MD Maastricht, Netherlands

[5] Department for Gynecology and Obstetrics, University Hospital Essen, University of Duisburg-Essen, Hufelandstr. 55, 45147 Essen, Germany

Esther Schwich	Esther.Schwich@uk-essen.de
Vera Rebmann	Vera.Rebmann@uk-essen.de
Rafael Tomoya Michita	rafael.michita@gmail.com
Hana Rohn	Hana.Rohn@uk-essen.de
Jan Willem Voncken	W.Voncken@maastrichtuniversity.nl
Peter A. Horn	Peter.Horn@uk-essen.de
Rainer Kimmig	Rainer.Kimmig@uk-essen.de
Sabine Kasimir-Bauer	Sabine.Kasimir-Bauer@uk-essen.de
Paul Buderath	Paul.Buderath@uk-essen.de

*** Correspondence:**

Vera Rebmann

Institute for Transfusion Medicine
University Hospital Essen
University of Duisburg-Essen
Virchowstr. 179
45147 Essen
Germany
Phone: +49 201 723 4206
Vera.Rebmann@uk-essen.de

Supplementary Data 1. Linkage Disequilibrium (LD) of 15 analyzed polymorphic variations in the HLA-G 3'UTR. The LD pattern was evaluated by calculating r^2. High pairwise LD (r^2) between variants is illustrated with dark shading. The r^2 values (×100) for the marker pairs are listed in the corresponding boxes. White color indicates r^2=0, shades of grey indicate $0<r^2<1$, and black color indicates r^2=1.

All individuals

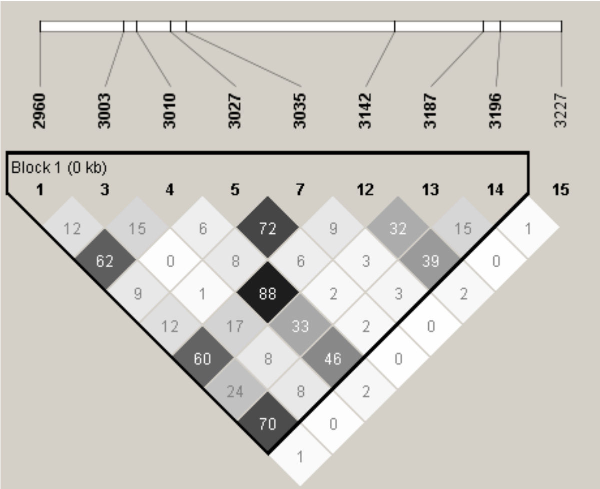

Cases only

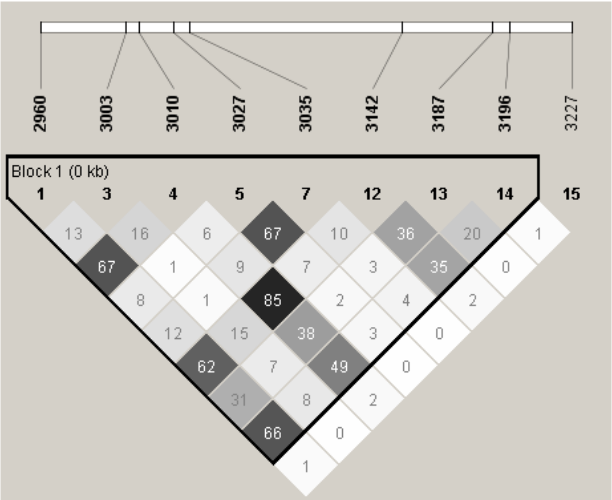

Controls only

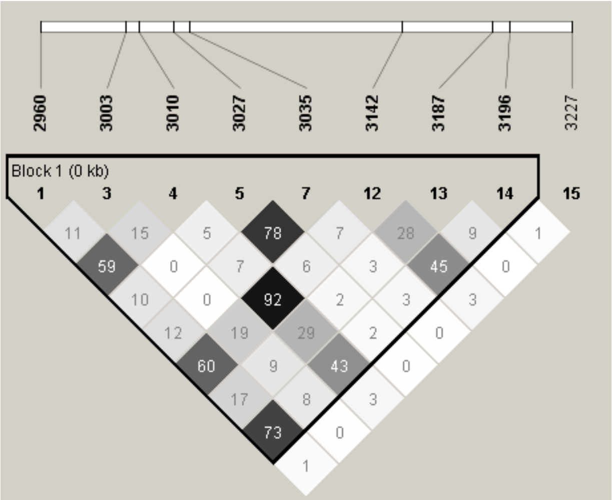

Supplementary Data 2. Haplotype frequencies of polymorphic sites detected at the *HLA-G* 3'UTR in epithelial ovarian cancer patients (n=79) and healthy donors (n=75).

Haplotype	Frequency total	Frequency HD	Frequency EOC
**UTR-1**	0.2919	0.2864	0.2972
**UTR-2**	0.2880	0.2786	0.2969
**UTR-3**	0.0916	0.1144	0.0699
**UTR-4**	0.1591	0.1666	0.1519
**UTR-5**	0.0195	0.0133	0.0253
**UTR-6**	0.0255	0.0192	0.0315
**UTR-7**	0.0552	0.0533	0.0570
**UTR-18**	0.0292	0.0333	0.0253
**UTR-undes.**	0.0130	0.0000	0.0254

Haplotype phasing was assessed by PHASE 2.1 software using default parameters.

EOC – epithelial ovarian cancer; HD – healthy donors; undes. – undesignated (see **Additional File 1** for reference); UTR – untranslated region

Supplementary Data 3. Haplotype and genotype frequencies of the *HLA-G* 3'UTR in EOC patients and healthy donors.

	**HD** N=75 (%)	**EOC** N=79 (%)	p^a	OR (95% CI)
**UTR-1**				
pos	39 (0.52)	41 (0.52)		
neg	36 (0.48)	38 (0.48)	0.9900	1.004 (0.533 – 1.890)
Genotypes				
UTR-1/UTR-1	4 (0.05)	6 (0.08)		
UTR-1/UTR-X	35 (0.47)	35 (0.44)	0.8393	
UTR-X/UTR-X	36 (0.48)	38 (0.48)		
**UTR-2**				
pos	34 (0.45)	38 (0.48)		
neg	41 (0.55)	41 (0.52)	0.7308	0.895 (0.475 –1.686)
Genotypes				
UTR-2/UTR-2	4 (0.05)	9 (0.11)		
UTR-2/UTR-X	30 (0.40)	29 (0.37)	0.3990	
UTR-X/UTR-X	41 (0.55)	41 (0.52)		
**UTR-3**				
pos	16 (0.21)	10 (0.13)		
neg	59 (0.79)	69 (0.87)	0.1509	1.871 (0.789 – 4.437)
Genotypes				
UTR-3/UTR-3	1 (0.01)	0 (0.00)		
UTR-3/UTR-X	15 (0.20)	10 (0.13)	0.2620	
UTR-X/UTR-X	59 (0.79)	69 (0.87)		
**UTR-4**				
pos	24 (0.32)	21 (0.27)		
neg	51 (0.68)	58 (0.73)	0.4600	1.300 (0.648 – 2.608)
Genotypes				
UTR-4/UTR-4	1 (0.01)	2 (0.03)		
UTR-4/UTR-X	23 (0.31)	19 (0.24)	0.5884	
UTR-X/UTR-X	51 (0.68)	58 (0.73)		
**UTR-5**				
pos	2 (0.03)	4 (0.05)		
neg	73 (0.97)	75 (0.95)	0.4423	0.514 (0.091 – 2.892)
Genotypes				
UTR-5/UTR-5	0 (0.00)	0 (0.00)		
UTR-5/UTR-X	2 (0.03)	4 (0.05)	n.d.	
UTR-X/UTR-X	73 (0.97)	75 (0.95)		
**UTR-6**				
pos	3 (0.04)	8 (0.10)		
neg	72 (0.96)	71 (0.90)	0.1401	0.370 (0.094 – 1.451)
Genotypes				
UTR-6/UTR-6	0 (0.00)	1 (0.01)		
UTR-6/UTR-X	3 (0.04)	7 (0.09)	0.2858	
UTR-X/UTR-X	72 (0.96)	71 (0.90)		
**UTR-7**				
pos	7 (0.09)	9 (0.11)		
neg	68 (0.91)	70 (0.89)	0.6755	0.800 (0.282 – 2.272)
Genotypes				

UTR-7/UTR-7	1 (0.01)	0 (0.00)		
UTR-7/UTR-X	6 (0.08)	9 (0.11)	0.4662	
UTR-X/UTR-X	68 (0.91)	70 (0.89)		
**UTR-18**				
pos	5 (0.07)	4 (0.05)		
neg	70 (0.93)	75 (0.95)	0.6716	1.339 (0.3455 – 5.191)
Genotypes				
UTR-18/UTR-18	0 (0.00)	0 (0.00)		
UTR-18/X	5 (0.07)	4 (0.05)	n.d.	
UTR-X/UTR-X	70 (0.93)	75 (0.95)		
**UTR-undes.**				
pos	0 (0.00)	2 (0.03)		
neg	75 (1.00)	77 (0.97)	n.d.	
Genotypes				
UTR-?/UTR-?	0 (0.00)	2 (0.03)		
UTR-?/UTR-X	0 (0.00)	0 (0.00)	n.d.	
UTR-X/UTR-X	75 (1.00)	77 (0.97)		

CI – confidence interval; EOC – epithelial ovarian cancer; HD – healthy donors; n.d. – not determined; neg – negative; OR – odds ratio; pos – positive; undes. – undesignated; UTR – untranslated region; UTR-X – every other UTR

[a]p-values were calculated by GraphPad Prism using two-sided Chi-square test, alpha<0.05; OD, odds ratio

Supplementary Data 4. Individual analysis of eight SNPs[a] encompassing the *HLA-G* 3'UTR and their association with disease status and outcome.

Metastasis formation		M_1[c]	M_0[c]	*p*[b]	OR (95% CI)
**IN/DEL**	DEL/DEL	9	20	0.5990	1.43 (0.51 – 3.95)
	IN/DEL or IN/IN	12	38		
**+3003**	TT	17	40	0.3982	1.91 (0.56 – 6.50)
	CC or CT	4	18		
**+3010**	GG	10	15	0.0993	2.60 (0.92 – 7.37)
	CC or CG	11	43		
**+3027**	CC	20	50	0.4316	3.20 (0.38 – 27.28)
	AA or CA	1	8		
**+3035**	CC	20	46	0.1666	5.22 (0.63 – 42.9)
	TT or CT	1	12		
**+3142**	CC	9	17	0.2870	1.80 (0.64 – 5.08)
	GG or CG	12	41		
**+3187**	GG	5	1	**0.0044**	17.81 (1.94 – 163.7)
	AA or AG	16	57		
**+3196**	GG	4	7	0.4704	1.71 (0.45 – 6.59)
	CC or CG	17	51		
**+3227**	AA	0	4	0.5687	0.28 (0.01 – 5.46)
	GG or AG	21	54		
**Nodal status**		**pN_1[c]**	**pN_0[c]**	*p*[b]	OR (95% CI)
**IN/DEL**	DEL/DEL	12	5	0.7568	1.49 (0.42 – 5.20)
	IN/DEL or IN/IN	21	13		
**+3003**	TT	26	12	0.5024	1.86 (0.51 – 6.73)
	CC or CT	7	6		
**+3010**	GG	11	3	0.3259	2.50 (0.59 – 10.51)
	CC or CG	22	15		
**+3027**	CC	28	16	1.0000	0.70 (0.12 – 4.03)
	AA or CA	5	2		
**+3035**	CC	28	13	0.2957	2.15 (0.53 – 8.77)
	TT or CT	5	5		
**+3142**	CC	11	4	0.5268	1.75 (0.46 – 6.59)
	GG or CG	22	14		
**+3187**	GG	2	0	0.5341	2.94 (0.13 – 64.6)
	AA or AG	31	18		
**+3196**	GG	6	1	0.3979	3.78 (0.42 – 34.19)
	CC or CG	27	17		
**+3227**	AA	2	1	1.0000	1.10 (0.09 – 13.00)
	GG or AG	31	17		
**FIGO**		**III-IV[c]**	**I-II[c]**	*p*[b]	OR (95% CI)
**IN/DEL**	DEL/DEL	29	0	0.0802	8.618 (0.47 – 158.90)

	IN/DEL or IN/IN	44	6		
**+3003**	TT	53	4	0.6686	1.33 (0.22 – 7.81)
	CC or CT	20	2		
**+3010**	GG	25	0	0.1690	6.84 (0.37 – 126.30)
	CC or CG	48	6		
**+3027**	CC	65	5	0.5283	1.63 (0.17 – 15.72)
	AA or CA	8	1		
**+3035**	CC	64	2	**0.0058**	14.22 (2.27 – 89.16)
	TT or CT	9	4		
**+3142**	CC	26	0	0.1701	7.25 (0.39 – 134.00)
	GG or CG	47	6		
**+3187**	GG	6	0	1.0000	1.25 (0.06 – 24.84)
	AA or AG	67	6		
**+3196**	GG	11	0	0.5875	2.39 (0.13 – 45.48)
	CC or CG	62	6		
**+3227**	AA	4	0	1.0000	0.84 (0.04 – 17.44)
	GG or AG	69	6		
**CTC prior to therapy**		**CTC pos[c]**	**CTC neg[c]**	p^b	OR (95% CI)
**IN/DEL**	DEL/DEL	4	25	0.3854	0.49 (0.14 – 1.70)
	IN/DEL or IN/IN	12	37		
**+3003**	TT	14	43	0.2100	3.09 (0.64 – 14.98)
	CC or CT	2	19		
**+3010**	GG	3	22	0.2347	0.42 (0.11 – 1.63)
	CC or CG	13	40		
**+3027**	CC	15	54	0.6764	2.22 (0.26 – 19.20)
	AA or CA	1	8		
**+3035**	CC	15	50	0.2829	3.60 (0.43 – 30.01)
	TT or CT	1	12		
**+3142**	CC	4	22	0.5527	0.61 (0.17 – 2.10)
	GG or CG	12	40		
**+3187**	GG	0	6	0.3363	0.26 (0.01 – 4.93)
	AA or AG	16	56		
**+3196**	GG	6	5	**0.0075**	6.84 (1.75 – 26.76)
	CC or CG	10	57		
**+3227**	AA	2	2	0.1847	4.29 (0.55 – 33.12)
	GG or AG	14	60		
**DTC prior to therapy**		**DTC pos[c]**	**DTC neg[c]**	p^b	OR (95% CI)
**IN/DEL**	DEL/DEL	10	19	0.8101	0.83 (0.32 – 217)
	IN/DEL or IN/IN	19	30		
**+3003**	TT	23	34	0.4323	1.69 (0.57 – 5.00)
	CC or CT	6	15		
**+3010**	GG	9	16	1.0000	0.93 (0.35 – 2.49)
	CC or CG	20	33		
**+3027**	CC	27	42	0.4715	2.25 (0.43 – 11.65)
	AA or CA	2	7		

**+3035**	CC<br>TT or CT	25<br>4	40<br>9	0.7571	1.40 (0.39 – 5.06)
**+3142**	CC<br>GG or CG	11<br>18	15<br>34	0.6204	1.39 (0.53 – 3.64)
**+3187**	GG<br>AA or AG	2<br>27	4<br>45	1.0000	0.83 (0.14 – 4.86)
**+3196**	GG<br>CC or CG	8<br>21	3<br>46	**0.0156**	5.84 (1.40 – 24.27)
**+3227**	AA<br>GG or AG	2<br>27	2<br>47	0.6254	1.74 (0.23 – 13.08)
**CTC after to therapy**		**CTC pos**[c]	**CTC neg**[c]	***p***[b]	OR (95% CI)
**IN/DEL**	DEL/DEL<br>IN/DEL or IN/IN	3<br>7	8<br>10	0.6888	0.54 (0.10- 2.77)
**+3003**	TT<br>CC or CT	8<br>2	13<br>5	1.0000	1.54 (0.24 – 9.90)
**+3010**	GG<br>CC or CG	1<br>9	6<br>12	0.3642	0.22 (0.02 – 2.19)
**+3027**	CC<br>AA or CA	9<br>1	18<br>0	0.3571	0.17 (0.01 – 4.62)
**+3035**	CC<br>TT or CT	9<br>1	18<br>0	0.3571	0.17 (0.01 – 4.62)
**+3142**	CC<br>GG or CG	2<br>8	7<br>11	0.4170	0.39 (0.06 – 2.42)
**+3187**	GG<br>AA or AG	0<br>10	2<br>16	0.5238	0.31 (0.01 – 7.22)
**+3196**	GG<br>CC or CG	3<br>7	4<br>14	0.6744	1.50 (0.26 – 8.64)
**+3227**	AA<br>GG or AG	0<br>10	1<br>17	1.0000	0.56 (0.02 – 14.94)

CI – confidence interval; CTC – circulating tumor cells; DTC – disseminated tumor cells; M0 – no metastasis formation; M1 – metastasis formation; neg – negative; OR – odds ration; pos – positive

[a]*HLA-G* 3'UTR SNP at position +3001, +3032, +3052, +3092, +3111 and +3121 were not included in this overview as all subjects had the same genotype.

[b]p-values were calculated by GraphPad prism using Fisher's exact test, alpha<0.05

[c]numbers reflect cases
